# Some Account of a Fever Produced by the Decomposition of Potatoes, in the Village of Almont, Michigan

**Published:** 1853-09

**Authors:** F. K. Bailie


### SELECTIONS

FROM

### FOREIGN AND HOME MEDICAL JOURNALS.

---

*Some Account of a Fever produced by the Decomposition of Potatoes, in the Village of Almont, Michigan.* By F. K. Bailie, M D. It is proposed, in this communication, as well as may be, to give an account of a disease which commenced in this place in the autumn of 1846. Up to this time, from the first settlement of the town, the diseases in this region were of the same character as those usually met with in newly settled places in the West. The country is generally level, and well timbered with beach, maple, oak, etc.

During the summer of 1846, there was erected in this village a large building, to be used in the manufacture of potato starch. The farmers, within five miles in each direction, contracted to produce potatoes, and draw them to the factory. As soon as the middle of October, there were delivered and deposited in one reservoir, about thirty thousand bushels. The machinery not being in readiness as soon as was expected, this amount of potatoes laid some two or three weeks. The weather being warm, the whole mass soon began to rot. A small proportion of them were diseased when drawn, but they were generally in a sound condition. As soon as it could be done the pile was overhauled, and not for from twenty thousand bushels removed into the yard in front of the mill. This mass was some three or four rods in extent each way, and four or five feet deep in the centre. The smell emanating from this offensive heap of vegetable matter was almost intolerable,

The fevers during the summer and the first of the fall months had been of a milder type than usual, yielding to proper treatment in a few days. During the months of November and December a typhoid tendency could be perceived in every case that occurred. It was evident that some modifying cause was operating. One man, who was engaged in removing potatoes from the mill, was attacked with a remitting fever; but about the seventh day, congestion of the brain set in, and he soon died. [This case will be alluded to again ]

Another case was that of a young lady, who had spent the summer some six miles from the village, in a neighborhood where fevers of the common type were prevalent, and had during the time an attack of remittent, but partially recovered before returning home. In the fore part of November she was taken with a relapse, in which the disease assumed a typhoid form. [The case will also be referred to again.]

During the fore part of December many cases of fever occurred, assuming a continued form, and none of the common type. As I took no notes at the time, it will be impossible to give in detail all the appearances in any one case, but will endeavor to describe, in general, most of the symptoms characterizing a majority of the cases.

This disease seldom commenced abruptly with a chill, but the patient complained of an uneasy sensation in the head, back, and limbs. Many, in describing their feelings, said they thought they should have the ague. There were languor and lassitude, no distinct chilliness, but an inclination to draw up to the fire, and an indisposition to exertion either physical or mental. The tongue was found to be coated with a whitish fur, there was a disagreeable taste in the mouth, and the breath was fœtid. The state of things just described continued sometimes a week; but generally the disease became confirmed in five or six days. Usually, at the expiration of this forming stage, a chill, more or less distinct, would usher in the disease, after which no more coldness would be experienced. At other times, the alternate cold and heat would gradually give place to continued heat and excitement, rendering it difficult to ascertain the exact time to date the outset.

The author gives the following synopsis of the symptoms and appearances that characterised the disease—

1. The pulse was frequent and quick. With an occasional exception, it was not less than 100 in a minute, and frequently going as high as 140 or 150 in cases that recovered. It was sometimes full, but always easily compressed beneath the finger. In the worst cases, and in feeble constitutions, it was sometimes found to intermit, which was usually an unfavorable symptom.

2. The breathing was hurried, frequently so much so as to occasion panting

3. Morbid sweating was common, and was considered indicative of danger.

4. Diarrhea was almost unfailing, and in many instances very excessive. Sometimes its existence was coëval with the attack, but frequently induced by the action of cathartics taken by the patient before calling medical aid, which developed the disease, that otherwise might not have occurred.

5. Tympanites was an attendant generally where there was diarrhea, but sometimes when the bowels were torpid.

6. Cough was common, and frequently the first symptom complained of. The patient would seem to have a cold, cough a little for a day or two, when other symptoms supervened, and the disease was established.

7. Hemorrhage from the bowels occurred in some cases, to such an extent as to exhaust the patient in a few hours. Sometimes the blood flowed unmixed, but commonly attended by morbid secretions.

8. The nervous system was in a marked degree affected in every case, more or less. Subsultus was common in the comparatively mild cases—worse, however, in persons of a strictly nervous temperament. Inability to confine the attention, indifference to surrounding objects and their own condition was apparent. But little solicitude was manifested in respect to recovery.

9. Delirium was common in the most cases, in some of which the patient would be almost uncontrollable. In a majority of cases the patient was so stupid that it was difficult to obtain a correct answer to an inquiry. He might, perhaps, be aroused enough to answer one question, but would relapse immediately into a snoring sleep.

10. Deafness was almost unfailing, and to such a degree, in some cases, as to render it difficult to make the patient hear.

11. Retention of the urine was an occasional source of trouble, most common in females.

12. Forgetfulness of what had occurred during the disease was common in the severe cases, and in the mild the memory was confused. Unconsciousness of the lapse of time may be mentioned in this connection; and, if a patient was unable to tell at any time how long he had been sick, or could not remember the day of the week, the prognosis might be considered as doubtful.

13. The rose-colored eruption attended a sufficient number of cases to be considered as diagnostic.

14. Subsultus and hiccough also were common — the former in cases of every grade, but the latter only in the worst cases.

Respecting the nature of this disease there can be no doubt. The foregoing enumeration of symptoms render its peculiar character conclusive.

The following facts are given to show that the fever was more or less contagious —

The smell of the potatoes was noticed little or none in parts of the village directly north of the mill while N. E. and N.W. it was almost intolerable. The subjects were generally young persons from eight to thirty years of age. In some instances whole families were affected. Facts may be related which would lead us to consider the disease as contagious. About the time that Miss B (the young lady refferred to) died, a daughter of Mr. K., who lived in the house adjoining, was attacked. About the same time a sister of Miss B. was attacked, and died on the seventeenth day of the disease. A young lady (Miss F.) who assisted in the family of Mr. K., was also taken sick, and remained in the family. A son of Mr. K. was taken about ten days after the daughter, and Mrs. K. was attacked before the rest recovered. After the younger Miss B. died, and previous to the attack of the other, the family removed to a house across the street. The wife of the man who went into the house Mr. B. had left, was attacked about the same time. Her husband also had some of the symptoms but they were thrown off. Miss A., who went into the family of Mr. K. after Miss F. was taken sick, began to complain in about a week, and went home to an adjoining town ten miles distant. She had the disease and died. Three or four of her father's family were afterwards attacked, and a brother died. No other cases occurred in that neighborhood. A sister-in-law and niece of Mr. K., who had assisted considerably in the care of the sick in his family, had the disease, but in a mild form. They resided on Main street, out of the range of the miasm, and were not in Mr. M.'s house more than one-third of the time. Mr. K. had some of the premonitory symptoms, but the disease was not developed. A sister of Miss F., who was with her about four weeks, was attacked and went home. She had the disease, and a brother, who resided in the family, also took it from her and died. A yovng man who resided in the family was attacked, and before he recovered, his father, who resided about twenty-five miles south, came and took him home. After his arrival at his father's, some of the family was taken sick, and from thence the disease spread until the whole household (the parents and seven children) died; the one who was taken sick here alone escaping.

After the death of the second Miss B., her father, a sister, and three other members of the family, were attacked, and had a se ere form of the disease. Many others of the neighbors who had watched with these families, either had the disease or felt the symptoms.

After the opening of spring no cases occurred until September, when the disease reäppeared. In the main, it presented the same characteristics during the autumnal months as it had during the previous winter. The principal variation was a tendency to assume a periodical form after the crisis, there being a chill, succeeded by the hot and sweating stages, as in ordinary intermittents, and susceptible of being arrested by the prompt administration of anti-periodics.

In such cases there seemed to be, in addition to the local miasm, the usual general influence tending to produce intermittents; and the typhoid tendency predominating, and expending its force first, left the system a prey to a predisposition less potent. This fact goes to prove that more than one cause may be operating in the atmosphere at the same time, each in its turn producing its effect according to its power to do so.

Such results occurred in cases where there was no organic lesion in any part, but a debilitated condition of the system, produced by the effect of the continued form of fever. When this crisis arrived the system was in a state to convalesce; but the ordinary causes of intermittents being in operation, a paroxysm would come on as stated above. Recovery would follow rapidly after the periodic tendency was interrupted, as after ordinary intermittents.

Post mortem examinations were made in three cases. The first was that of the young lady first mentioned. There were ulcerations through the whole extent of the intestines, from the duodenum to the rectum, the deepest being in the small intestines. The ulcerated patches were from the diameter of two lines to that of a pin head. The intestines were found filled with black blood, and before death some quarts had passed off. She might be said to have died of hemorrhage, as the other symptoms had not been particularly unfavorable. She, it will be remembered, died in December, 1846.

The second was in the case of a little girl, four years of age, who died in the fall of 1848. She had had whooping cough for two or three weeks at the time the fever set in, and her death was caused by congestion of the brain. The brain was filled with blood, and the lungs also were considerably congested. The mucous membrane of the small intestines was inflamed to a considerable extent. There were small elevated spots upon the internal surface of the bowels, which were of a bright red. The case was an interresting one, from the fact that we had an opportunity of examining the condition of the entire mucous membrane at an early stage of the disease, which we could seldom have done, as the fever was not sufficient cause to produce death. There was much more disease than any external sign indicated, from which we might infer that in all cases there was inflammation to a greater or less extent.

The third case was that of a young man, twenty-two years of age, who died in the autumn of 1847. He had resided during the summer in St. Joseph's county, and had suffered while there from a remittent fever. At the time he was attacked with continued fever, he had not recovered from his former sickness, but was able to be about the streets. He was not as sick to appearance as many others had been who recovered. He died on the twehty-second day from the time of attack. Half an hour before his death he asked to be helped into a chair, was cheerful, spoke of his hopes of recovery, and took some food. He very soon complained of a severe pain in the abdomen, and a deathly faintness. Stimulants were administered, but he soon sunk and died in the chair. In this case there were extensive ulceratioes of the small intestines throughout the whole extent. About a foot of the ileum was gangrenous, with perforations in two or three places, the largest of which was an inch and a half in length. The intestines were diseased through the whole length to some degree, presented a livid appearance, and considerable effusion had taken place in the abdomihal cavity. He had strength enough to walk across the room within five days of his death.—*North-Western Medical and Surgical Journal.*

